# Mechanistic Links Between the Gut Microbiome and Longevity Therapeutics

**DOI:** 10.3390/biomedicines14020316

**Published:** 2026-01-30

**Authors:** Noelia Garzon-Escamilla, Miriam Medina-Cardena, Preeti Roy, Jessica Trent, Joud Jamous, Yalini Somesan, Sandy J. Denslow

**Affiliations:** Department of Molecular Biosciences, University of South Florida, Tampa, FL 33620, USA; noeliag@usf.edu (N.G.-E.); medinacardena@usf.edu (M.M.-C.); preetiroy@usf.edu (P.R.); jgrafius@usf.edu (J.T.); joudjamous@usf.edu (J.J.); yalinis@usf.edu (Y.S.)

**Keywords:** gut microbiome, aging, metformin, senolytics, GLP-1, SGLT2i, rapamycin, anti-inflammatories, sirtuins, spermidine

## Abstract

Aging is a multifactorial biological process marked by the progressive decline in cellular and physiological functions, increasing susceptibility to chronic diseases and mortality. Recent research has identified the gut microbiome as a key modulator of aging, influencing immune regulation, metabolic homeostasis, and neuroendocrine signaling. A diverse and balanced gut microbiota promotes healthspan by supporting gut barrier integrity, nutrient metabolism, and anti-inflammatory responses, whereas dysbiosis contributes to the onset and progression of age-related diseases, including neurodegeneration, cardiovascular conditions, cancer, and metabolic disorders. Currently, anti-aging interventions targeting key aging pathways, such as insulin/IGF-1 signaling, mTOR, AMPK, and sirtuins, are a major focus in the field of geroscience. Compounds such as metformin, rapamycin, anti-inflammatories, GLP-1 agonists, senolytics, spermidine, SGLT2 inhibitors, and sirtuin activators have shown lifespan extension in animal models. In humans, some of these interventions are associated with improvements in healthspan-related outcomes, including metabolic, cardiovascular, musculoskeletal, respiratory, cognitive and ocular functions. Notably, the gut microbiome may serve as both a mediator and modulator of these interventions, influencing drug metabolism, efficacy, and host responses. This review synthesizes current evidence on the gut microbiome’s role in aging, examining its role as both mediator and modulator of longevity interventions and how microbiome-associated mechanisms intersect with emerging anti-aging therapeutics.

## 1. Introduction

Aging is an intricate process characterized by a gradual decline in biological functions, leading to an increased susceptibility to diseases and death [1]. The progressive deterioration of cellular and molecular structures results in a diminished capacity of organs and tissues to repair themselves, contributing to an overall decline in the organism [1,2]. In recent years, scientists have established the hallmarks of aging that represent the underlying biological mechanisms contributing to aging and age-related diseases. Genomic instability, telomere attrition, epigenetic alterations, loss of proteostasis, disabled macroautophagy, deregulated nutrient-sensing, mitochondrial dysfunction, cellular senescence, stem cell exhaustion, altered intracellular communication, chronic inflammation, and dysbiosis represent the twelve updated hallmarks of aging currently recognized by the geroscience community [3]. In addition, genetic, environmental, and lifestyle factors have been shown to influence these hallmarks, subsequently influencing how we age [3,4]. Scientists are exploring innovative approaches in the effort to slow aging and prevent age-related diseases, with much of the attention focused on the gut. It is known that the intestine hosts a highly complex and diverse community of living microorganisms, also referred as the gut microbiota [5,6]. The dynamic interaction between our gut microbiome and our body is a key factor in the aging process, significantly influencing our susceptibility to develop age-related diseases [7]. A diverse and balanced microbiota supports immune function, facilitates nutrient absorption and digestion, promotes the production of beneficial short chain fatty acids (SCFAs) and vitamins, maintains gut barrier integrity, and prevents the colonization of harmful pathogens [6,8], all of which are associated with optimal health in aging individuals. On the other hand, dysbiosis is an imbalance of the gut microbiota that can exacerbate aging-related diseases by triggering inflammation, disrupting immune and metabolic homeostasis, and weakening gut barrier integrity [7,9,10,11]. In fact, certain microbiome profiles shared across multiple model organisms have been linked to specific age-associated pathologies such as neurodegenerative diseases, cardiovascular diseases, cancer, and metabolic disorders [6,8,12]. While individual variability and environmental influences currently limit the use of microbiome profiles as standalone diagnostic tools, accumulating evidence suggests that microbiome changes may reflect underlying aging-related processes and could potentially serve as supplementary biomarkers for the early detection of age-related diseases.

The gut microbiome plays an essential role in regulating multiple biological functions through a complex network of neural, endocrine, and immune signals collectively referred as the gut connectome [8,13,14]. Studies have shown that the gut microbiota interacts with this connectome via microbial-associated molecular patterns (MAMPs), microbial metabolites, and microbe-derived neurochemicals [8,9]. MAMPs are molecular signatures including lipopolysaccharides, peptidoglycans, flagellins, or specific bacterial nucleic acids that can indirectly influence the functions of the gut connectome [8]. For example, bacterial lipopolysaccharide (LPS) activates Toll-like receptor 4 (TLR4) signaling, promoting systemic inflammation and altering vascular and neural signaling. On the other hand, gut bacteria can produce microbial metabolites such as SCFAs, indole derivatives, trimethylamine-N-oxide (TMAO) and polyamines; as well as several neurotransmitters including serotonin, GABA, or acetylcholine that can impact the immune, endocrine, and neural signaling of the host [8,9]. Notably, the SCFA butyrate has been shown to enhance blood–brain barrier integrity and modulate microglial activation, linking microbial metabolism to central nervous system function. These bacterial metabolites can influence cardiovascular health by modulating inflammation, blood pressure, cholesterol levels, and atherosclerosis risk [15,16,17,18]. They also affect metabolic disorders by altering insulin sensitivity, GLP-1 secretion, lipid metabolism, bile acid production, and fat storage [19,20,21,22,23]. In addition, studies indicate that the gut microbiome affects DNA damage, cancer cell metabolism, and inflammatory signaling, thereby promoting pro-tumorigenic environments [24,25,26,27]. Moreover, microbial-mediated factors have been shown to impact the integrity of the blood–brain barrier, which regulates the exchange of molecules between the bloodstream and the brain [9]. Taken together, this evidence highlights the wide-ranging effects of the gut microbiota on multiple biological functions, underscoring its significant role in health and disease. In this review, we synthesize current knowledge on the role of the gut microbiota in the aging process and evaluate how other emerging anti-aging interventions, including pharmacological agents, reshape microbial composition and function. By identifying convergent pathways and overlapping microbial signatures, this work seeks to clarify whether microbiota-targeted and drug-based strategies act through common mechanisms to promote aging.

## 2. Methodology

This narrative review synthesizes the current knowledge on gut microbiome contributions to aging and examines whether emerging anti-aging interventions reshape microbial composition and function through shared microbial and host pathways. Literature for this review was identified through PubMed, Google Scholars, Clinicaltrials.gov, and ScienceDirect using a combination of keywords related to gut microbiota, age-related diseases and anti-aging interventions. Studies were selected based on relevance to microbiome–aging interactions and the effects of interventions on microbial composition or function. Review articles from 2017 to 2025 were included alongside primary research articles addressing the central objective. Data were gathered separately for mechanistic effects and microbial changes, organized by bacterial phylum or genus/species, categorized by host organism, and, for humans, classified by health status or age-related condition to enable clearer interpretation. Findings were synthesized thematically, focusing on convergent microbial signatures, shared host pathways, and outcomes linked to delayed aging and improved healthspan. Unless otherwise stated, evidence for lifespan extension is derived primarily from in vitro and animal studies, whereas human data largely reflect disease-specific or healthspan-associated outcomes rather than direct effects on aging or longevity. Literature was searched through December 2025.

## 3. Gut Microbiota and Aging

**Figure 1:**
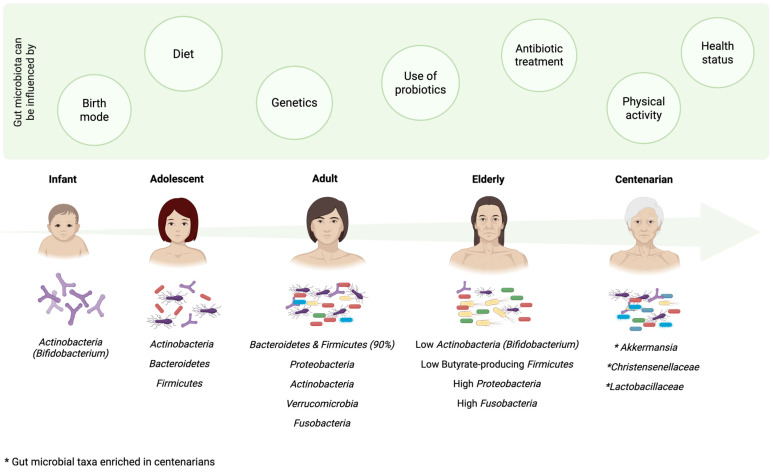
Host and environmental drivers of the gut microbiome dynamics across aging. The gut microbiome undergoes dynamic changes across the human lifespan, shaped by host factors and lifestyle. These influences drive age-dependent shifts in microbial diversity and function, impacting immune regulation, metabolic homeostasis, and susceptibility to age-related diseases. Asterisks indicate gut microbial taxa enriched in centenarians, suggesting a possible association with exceptional longevity. Created in BioRender. Garzon-Escamilla, N. (2026) https://BioRender.com/q9r6r70.

The human gut microbiome undergoes profound changes throughout life, shaped by host biology as well as environmental and lifestyle factors (Figure 1). After birth, the gut microbiome develops as a transitional ecosystem dominated by *Actinobacteria*, especially *Bifidobacterium*. [28,29,30]. In this critical window, factors such as mode of delivery, whether vaginal or cesarean, and early feeding practices help shape the foundation for microbial diversity and stability, with lasting implications for immune development, metabolism, and disease susceptibility [30]. Notably, a *Bifidobacterium*-rich environment is crucial for the maturation of the infant immune system [31]. Diet diversification during early childhood drives dynamic shifts in the gut microbiota, with *Actinobacteria*, *Bacteroidetes*, and *Firmicutes* predominating [10,11]. This composition continues to evolve through adolescence, in which *Actinobacteria* begin to decline [10,11,32]. Over time, the gut microbiome transitions toward an adult-like state, predominantly composed of *Firmicutes* and *Bacteroidetes*, which together account for approximately 90% of the microbial community [10,11,33]. Other phyla, including *Proteobacteria*, *Actinobacteria*, *Verrucomicrobia*, and *Fusobacteria* are present at lower abundances [10]. After the age of 70, the gut microbiota diversity and stability decline significantly, characterized by reductions in *Actinobacteria*, especially *Bifidobacterium*, and beneficial butyrate-producing *Firmicutes*, alongside an increase in *Proteobacteria* and *Fusobacteria*, the latter known for potential pathobiont activity [10,11,32,34]. Centenarians offer a unique perspective on how gut microbiota may contribute to healthy aging. Certain beneficial *Verrucomicrobia*, especially in *Akkermansia* spp., and *Firmicutes* family including *Christensenellaceae* and *Lactobacillaceae* are enriched in centenarian and supercentenarian populations from China, Italy, and female individuals from India [11,32,34]. These bacteria have been associated with reduced systemic and intestinal inflammation, improved gut barrier integrity, enhanced immune modulation, and improved insulin sensitivity [34,35,36,37,38,39]. These effects have been consistently shown to promote healthier aging by preventing or ameliorating metabolic diseases such as type 2 diabetes (T2D), obesity, liver diseases, colitis, and inflammatory bowel disease [37,38,40,41]. In summary, microbiota diversity peaks within the first three years of life, remains relatively stable throughout adulthood, and considerably diminishes after the age of 70 [10], potentially contributing to the onset of age-related diseases. Additionally, it is important to note that factors like host genetics, probiotic and antibiotic use, exercise and overall health status can influence the gut microbiome across the lifespan [7,42,43,44,45].

Several studies agree on the fact that a healthy microbiome is characterized by diversity, balance, and stability [6,10,46,47]. Having a wide range of microbial species in the gut enhances the microbiota’s resilience, which is essential for adaptation to environmental changes and stressors. Microbial variety allows the maintenance of essential functions such as digestion, immune responses, protection against pathogens, or nutrient production by constituting a robust and competent gut microbiome [10,46,48].

In addition to the importance of having a diverse and stable gut microbiome, numerous studies highlight the need to maintain a balanced intestinal microbiota, with particular attention to the *Firmicutes*-*Bacteroidetes* ratio. An increase in the *Firmicutes*-to-*Bacteroidetes* ratio is associated with an increased production of SCFAs [10,49]. The primary SCFAs associated with gut health, including butyrate, acetate and propionate are produced by gut microbiota in the colon. These SCFAs are among the most-studied bacterial metabolites due to their abundance and effects on host physiology such as gut motility, gut barrier integrity maintenance, anti-inflammatory effects, and modulation of the neuro-immunoendocrine function [49,50,51,52,53]. However, an imbalanced production of SCFAs has also shown to influence energy harvest and storage and subsequently contribute to different metabolic disorders such as obesity and T2D [10,49,54]. Thus, while SCFAs play important roles in host physiology, their dysregulation may shift their effects from beneficial to detrimental. Therefore, maintaining an equilibrium in SCFA production, which depends on both microbial balance and the health condition of the host, is a critical determinant of whether their impact is protective or counterproductive.

## 4. Effects of Anti-Aging Therapies on Gut Microbiome Composition

**Figure 2:**
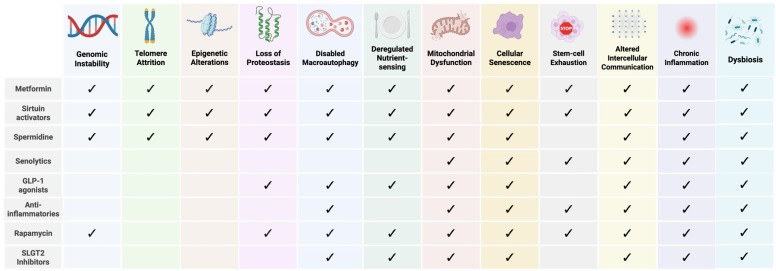
Impact of anti-aging interventions on hallmarks of aging. Summary of the reported impacts of the main geroprotective drugs on the twelve hallmarks of aging. Checkmarks indicate interventions for which there is strong to moderate evidence of modulation, either direct or indirect, reflecting the diverse mechanisms through which these drugs may support healthy aging and tissue homeostasis. Created in BioRender. Medina-Cardena, M. (2026) https://BioRender.com/vjarxz7.

Over the past decades, anti-aging compounds such as metformin, sirtuin activators, spermidine, senolytics, GLP-1 agonists, anti-inflammatories, rapamycin, and SGLT2 inhibitors have emerged as a captivating focus of research, with hopes of extending both lifespan and healthspan [6,12,55]. These drugs primarily act by modulating hallmarks of aging (Figure 2) [12,55,56,57,58,59,60,61,62], strongly supporting the “Geroscience Hypothesis,” which posits that targeting fundamental aging processes could simultaneously mitigate multiple age-related diseases and promote healthy aging [63,64]. In fact, many of these drugs are currently undergoing clinical trials for their potential to prevent or even reverse aspects of aging [12]. Considering the microbiome’s pivotal role in regulating immune function, metabolic processes, and inflammation, as well as its ability to influence drug metabolism and efficacy [65], understanding whether longevity drugs affect the microbiome can offer new insights into their mechanisms of action and help refine anti-aging therapies.

## 5. Anti-Aging Drugs

### 5.1. Metformin

Metformin, a guanidine derivative, was first recognized in the 1920s for its ability to regulate blood glucose levels, and it is now one of the most widely prescribed medications for the treatment of T2D [66]. It benefits diabetic patients primarily by enhancing insulin sensitivity, stimulating glycolysis, and suppressing hepatic gluconeogenesis [12,57,67]. However, recent studies indicate that metformin exerts a much broader range of effects beyond glucose regulation, including cardiovascular protection, anti-tumorigenic properties, and general anti-aging benefits, increasing the survival rate among patients with diabetes and cardiovascular disease [12,57,58,68,69]. Moreover, metformin may promote longevity by reducing frailty in older diabetic patients and lowering the risk of cognitive decline [57,70]. Its diverse effects on lipid metabolism, mitochondrial function, inflammation, and cellular survival and proliferation [12,57,58,67,68,69] are partially modulated by epigenetic alterations that influence the expression of genes involved in energy homeostasis and stress resistance [71]. Collectively, these multifaceted actions of metformin underscore its potential as a geroprotective drug, improving not only metabolic health but overall lifespan and healthspan.

#### 5.1.1. Mechanisms of Action of Metformin

**Figure 3:**
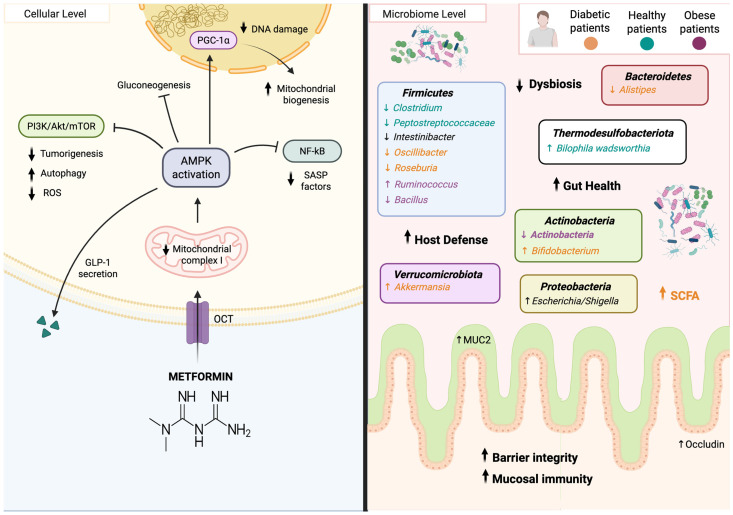
Overview of the pleiotropic effects of metformin at the cellular and microbiome levels. At the cellular level, metformin suppresses mitochondrial complex 1, leading to AMPK activation. Downstream effects include reduced gluconeogenesis, modulation of PI3KAkt/mTOR and NF-κB signaling, decreased SASP factors and oxidative stress, enhanced autophagy, mitochondrial biogenesis, and GLP-1 secretion. In parallel, metformin reshapes gut microbiota, reducing dysbiosis, and promoting gut health. Microbiome changes observed in diabetic patients are shown in orange, healthy individuals in blue, and obese patients in purple. Despite distinct microbial profiles across patient metabolic states, a shared decrease in *Intestinibacter* and increase in *Escherichia* are observed. Created in BioRender. Garzon-Escamilla, N. (2026) https://BioRender.com/5qbecpw.

Several mechanisms have been proposed to explain the diverse effects of metformin in the human body (Figure 3). One well-established pathway suggests that metformin enters the cell via organic cation transporters (OCT) [72] and acts as an insulin sensitizer by mildly inhibiting the mitochondrial respiratory complex I, leading to reduced ATP synthesis and activation of AMPK [12,70,73]. Once activated, AMPK decreases hepatic glucose production in insulin-resistant diabetic patients and promotes mitochondrial biogenesis, thereby enhancing insulin sensitivity [12]. Furthermore, metformin can modulate glucose metabolism by increasing the secretion of GLP-1 in the gut, which stimulates insulin production and inhibits glucagon release [74,75]. On the other hand, AMPK activation indirectly suppresses the PI3K/AKT/mTOR pathway, contributing to the anti-tumorigenic effects by inhibiting cellular growth and proliferation [76,77], enhancing proteostasis through increased autophagy [76,77], and preserving genomic integrity by reducing oxidative stress and DNA damage [78]. AMPK activation also modulates PGC-1a, promoting mitochondrial biogenesis and antioxidant defense, which together minimizes telomere attrition and maintains chromosomal stability [79]. Interestingly, metformin’s ability to decrease DNA damage and promote metabolic health has been shown to support stem cell regeneration and restore CNS remyelination in aged animals [80]. Moreover, metformin exerts anti-inflammatory effects by suppressing NF-κB, reducing the secretion of pro-inflammatory cytokines, and reducing the expression of various senescence-related factors in multiple cell types, including endothelial cells, fibroblasts, adipocytes, and murine macrophages [70,81,82].

#### 5.1.2. Impact of Metformin on Gut Microbiota

Metformin-induced changes in the gut microbiome have been consistently observed (Figure 3) [83,84,85,86,87,88,89,90,91,92,93,94]. On the one hand, metformin has been reported to increase the expression of tight junction proteins, such as occludin, as well as mucin-2, the main component of intestinal mucus that contributes to gut barrier integrity and intestinal homeostasis [95]. By reinforcing barrier integrity, these effects reduce colonic inflammation, limit gut permeability, and prevent lipopolysaccharide translocation. On the other hand, there are inconsistencies in the reported effects of metformin on human gut microbiota, reflecting diverse findings between diabetic, obese, and healthy patients. Despite differences in study design and analytical approach, clinical trials consistently reveal shared microbial shifts in T2D patients treated with metformin, including increased abundances of *Escherichia*, *Akkermansia*, and SCFA-producing bacteria such as *Bifidobacterium* and *Butyrivibrio*, alongside decreased abundances of *Intestinibacter*, *Oscillibacter*, *Roseburia* and *Alistipes* [87,91]. Despite differences in study design and analytical approach, clinical trials consistently reveal shared microbial shifts in T2D patients treated with metformin, including increased abundances of *Escherichia*, *Akkermansia*, and SCFA-producing bacteria such as *Bifidobacterium* and *Butyrivibrio*, alongside decreased abundances of *Intestinibacter*, *Oscillibacter*, *Roseburia* and *Alistipes* [84,87,91,93]. On the other hand, studies in obese patients show a more heterogeneous set of microbial shifts after metformin administration. Two studies highlight an increase in *Escherichia*/*Shigella*, while other, more context-dependent changes include an increase in *Ruminococcus* and decreased abundances in *Intestinibacter*, *Roseburia*, *Actinobacteria*, and *Bacillus* [85,88,89,94]. In healthy individuals, metformin has been associated with a common increase in *Escherichia/Shigella*, together with reduced levels of *Clostridium*, and *Intestinibacter* [83,86]. More context-dependent findings include an increase in *Bilophila wadsworthia* and a decrease in *Peptostreptococcaceae.* Overall, the data indicate a consistent increase in *Escherichia* and a decrease in *Intestinibacter* following metformin treatment, regardless of the patient’s health status or study design. However, given the limited information on which *Escherichia* strains are affected and the role of *Intestinibacter* in gut health, it remains challenging to determine whether these microbial changes represent side effects or are part of a mechanistic pathway underlying metformin’s therapeutic action. The diversification across studies highlights the complexity of standardizing metformin’s impact on the gut microbiota, as its effects may vary depending on factors such as baseline microbial composition and host health status. To better understand these effects, more research is needed to clarify the role of *Intestinibacter* in human health, identify the specific *Escherichia* strains altered by metformin, and optimize treatment strategies to standardize its administration and effects across patients with different health conditions.

### 5.2. Rapamycin

Rapamycin was originally isolated as an antifungal metabolite produced by *Streptomyces hygroscopicus* from a soil sample collected on Easter Island, also known as Rapa Nui. However, once rapamycin was shown to inhibit the proliferation of eukaryotic cells, research shifted toward investigating its immunosuppressive and anticancer potential [96]. Studies revealed that rapamycin suppresses cell growth by inhibiting the target of rapamycin (TOR) in yeast [97], a protein later shown to be conserved in mammals, where it is called mTOR [98,99,100]. Various rapamycin analogs, known as rapalogs, including temsirolimus and everolimus, have been FDA-approved for the treatment of cancers such as urothelial, anal, and skin carcinomas, due to their potent anti-proliferative effects [98,99,100]. Moreover, rapamycin has been shown to promote longevity across various model organisms, including yeast, *Drosophila*, and *C. elegans* [101,102,103], and became the first pharmaceutical drug to significantly extend murine lifespan. [104]. Beyond its lifespan and antitumorigenic effects [105], rapamycin has shown benefits for cardiovascular [106,107,108] and neurological health [106,107,108]. Additionally, rapamycin has been used as a potent immunosuppressive drug to prevent allograft rejection in heart, liver, lung, and kidney transplants [109,110,111]. Altogether, rapamycin has gained recognition as a powerful agent not only for its anti-proliferative properties but also for its potential to promote longevity and support healthy aging.

#### 5.2.1. Mechanisms of Action of Rapamycin

**Figure 4:**
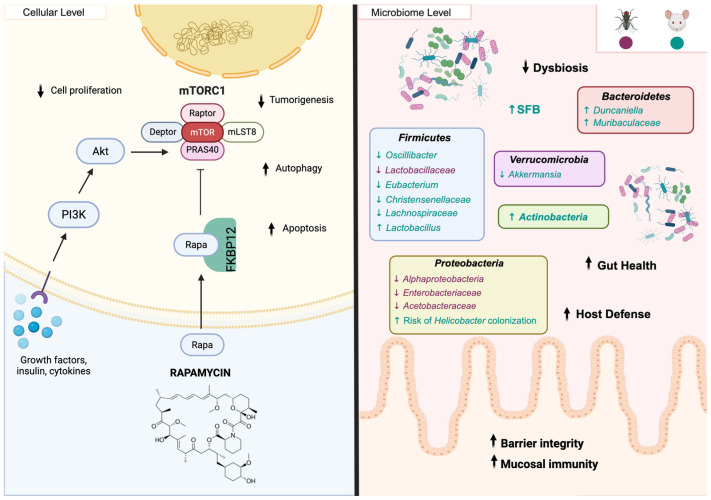
Overview of the pleiotropic effects of rapamycin at the cellular and microbiome levels. At the cellular level, rapamycin inhibits mTORC1 downstream of PI3K–Akt signaling, leading to reduced cell proliferation and tumorigenesis, with increased autophagy and apoptosis. In parallel, rapamycin remodels gut microbial composition in animal models. Microbial shifts observed in flies after rapamycin treatment are shown in purple while those identified in mice are shown in blue. These microbiome changes are associated with improved barrier integrity, enhanced mucosal immunity, and increased host defense. Created in BioRender. Roy, P. (2026) https://BioRender.com/olbxq5r.

Rapamycin acts by directly targeting mTOR (Figure 4), a serine/threonine protein kinase activated downstream of PI3K/Akt signaling and modulated by extracellular cues such as growth factors, nutrients, energy availability, and cytokines to regulate cell metabolism, growth, and proliferation [112,113]. mTOR assembles into two distinct complexes: mTORC1 and mTORC2 [100,114,115]. While mTORC1 regulates cell differentiation, growth, and RNA and protein synthesis [116]; mTORC2 is involved in cell proliferation, survival, and cytoskeletal organization [117]. Rapamycin binds to the 12 kDa FK506-binding protein (FKBP12) to form a complex that selectively inhibits mTORC1 activity, being the only mTOR complex immediately responsive to rapamycin inhibition [116]. Because mTORC1 inhibition is believed to mediate most of rapamycin’s lifespan-extending effects, research has primarily focused on this complex [118,119]. Accordingly, this review will also center on mTORC1. Rapamycin contributes to cancer prevention through both direct and indirect mechanisms. Directly, it inhibits mTORC1 in pre-cancerous and cancer cells, suppressing their growth and proliferation [105]. Indirectly, rapamycin limits angiogenesis [120] and induces autophagy and apoptosis [121], reducing the environmental conditions that promote tumor development. The anti-aging effects attributed to rapamycin arise from its ability to counteract the decline in cellular maintenance pathways driven by hyperactive mTORC1 signaling, particularly impaired proteostasis and reduced autophagy [122]. Rapamycin-induced autophagy has been shown to confer protection across multiple cell types and disease contexts. Specifically, it protects cardiomyocytes from apoptosis through suppression of endoplasmic reticulum stress [106], ameliorates Alzheimer’s-like behavioral deficits and restores synaptic plasticity by promoting the clearance of damaged mitochondria [110], reduces cellular senescence in cells undergoing endothelial-mesenchymal transition [123], and improves satellite cell differentiation [124]. Moreover, mTORC1 inhibition by rapamycin has been shown to restore non-canonical open reading frame translation in aging mice [125], contributing to improved protein homeostasis. On the other hand, chronic rapamycin treatment, which inhibits both mTORC1 and mTORC2 activity, has shown adverse effects including disruption of insulin signaling and lipid metabolism, ultimately promoting insulin resistance [126,127,128]. Although treatments such as metformin can counteract some of these adverse metabolic effects [128], this still highlights a major limitation of long-term rapamycin treatment as a therapeutic option. While research has focused on characterizing the molecular targets and pathways of rapamycin, its impact on the gut microbiome, an emerging regulator of aging and many age-related diseases, remains poorly understood. Given the microbiome’s important role in the aging process, we next aim to explore how rapamycin may influence both its composition and function.

#### 5.2.2. Impact of Rapamycin on Gut Microbiota

While research in humans is limited, compelling evidence suggests that rapamycin also reshapes the gut microbiome composition and abundance in flies and mice (Figure 4) [6,129,130,131]. A previous study showed that dietary rapamycin can slow down and reduce the growth of gut bacteria linked to age-related dysplasia in flies, including *Enterobacteriaceae*, *Lactobacillaceae*, and *Acetobacteraceae* [132]. Interestingly, rapamycin also lowered *Alphaproteobacteria* load in *Drosophila*, a bacterial group previously associated with increased mortality and declining health in the elderly [133]. Rapamycin treatment in adult flies delayed the onset of intestinal barrier dysfunction and mitigated age-associated microbial dysbiosis, improving gut health later in life [130]. Although rapamycin has been shown to induce changes in the composition of the fly gut microbiome, these shifts have not been associated with its lifespan-extending effects [130]. Furthermore, it was found that reassociating germ-free flies with the microbiota of conventional flies treated with rapamycin had no effect on host lifetime, indicating that rapamycin-induced microbiota alterations alone are insufficient to transfer longevity benefits [130]. On the other hand, transient rapamycin therapy in middle-aged mice led to a marked increase in the predominance of segmented filamentous bacteria (SFB) such as *Arthromitus* in the small intestine [134]. SFB is known to induce increased accumulation of T Helper 17 cells in the small intestine which plays an important role in mediating host defensive mechanisms to various extracellular bacterial infections [135]. Changes in the *Bacteroidetes* to *Firmicutes* ratio have been reported in mice following rapamycin treatment, characterized by a significant increase in *Bacteroidetes*, particularly in *Duncaniella* and *Actinobacteria* species, and a decrease in *Firmicutes*, including *Lachnospiraceae*, *Oscillibacter*, *Christensenellaceae*, and *Eubacterium* [129,131]. Interestingly, some of these bacterial shifts may be dose dependent [129]. In addition to altering microbial composition, rapamycin reprograms amino acid metabolism within the gut lumen and induces shifts in intestinal immune responses [131]. Rapamycin also ameliorated colitis in the mice microbiome by increasing beneficial bacteria such as *Lactobacillus* and reducing potentially harmful species associated with gut dysbiosis [136]. On the other hand, it has been suggested that rapamycin treatment can promote the colonization by *Helicobacter*, which may contribute to disease development, and to decrease *Akkermansia*, which has been previously mentioned to strengthen the gut barrier integrity [129]. Finally, mTORC1 inhibition restores goblet cells and Paneth cells, supporting the intestinal barrier, microbial balance, and mucosal immunity [129]. Together, these studies suggest that rapamycin treatment leads to beneficial effects in the gut of both mice and flies. However, these improvements appear to be independent of rapamycin’s longevity effects. Although these findings are promising, there is limited data available on the influence of rapamycin on gut health in humans. Therefore, additional studies are needed to clarify dose-dependent effects of rapamycin in the gut microbiota, how these microbial shifts translate to humans, and how they ultimately influence healthspan.

### 5.3. Senolytics

Senolytics research emerged around 2004, driven by the hypothesis that selectively targeting and removing senescent cells could alleviate age-related diseases [64,137]. Senescence-associated cell cycle arrest is primarily controlled by the p53/p21 and p16/RB tumor suppressor pathways [138,139,140], which inhibit cell proliferation to prevent the replication of damaged or potentially cancerous cells [139]. This irreversible arrest triggers the secretion of inflammatory cytokines, chemokines, growth factors, and proteases, altogether known as the senescence-associated secretory phenotype (SASP) [137,138,139]. When chronically active, SASP disrupts healthy tissue by promoting the accumulation of senescent cells in tissues, ultimately contributing to detrimental outcomes such as neurodegenerative diseases, atherosclerosis, and osteoarthritis [138,139,141]. Accumulating evidence shows that senescent cells directly impair physical dysfunction and reduce lifespan in mice [64,137,142], and transplanted senescent cells can spread senescence to neighboring cells and induce systemic physical decline in a dose-dependent manner [141,142]. Although no senolytic drugs have yet been FDA-approved for age-related diseases, the natural flavonoid quercetin (Q) and the anticancer targeted therapy dasatinib (D) have been repurposed and tested in combination in early human trials, where they show potential to reduce senescent cell burden and potentially ameliorate disorders such as Alzheimer’s disease, osteoporosis, and diabetes, reinforcing their potential for addressing age-related disorders [63,64,140,143,144,145,146].

#### 5.3.1. Mechanisms of Action of Senolytics

**Figure 5:**
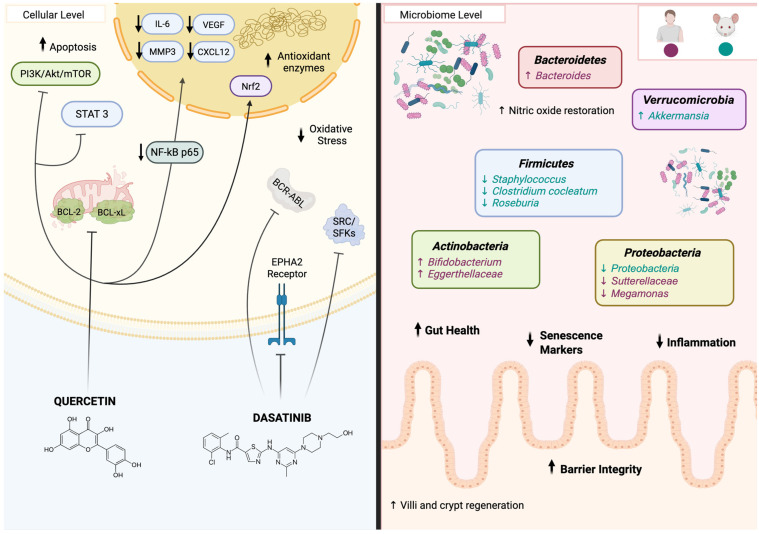
Overview of the pleiotropic effects of senolytics at the cellular and microbiome levels. Quercetin and dasatinib modulate key pathways involved in cellular senescence and inflammation, including PI3K/Akt/mTOR, STAT3, NF-κB, and Nrf2, promoting selective apoptosis of senescent cells and enhancing antioxidant defense. At the microbiome level, senolytic treatment induces compositional shifts in mice and human models associated with improved nitric oxide homeostasis, enhanced intestinal barrier integrity, increased villus and crypt regeneration, and overall reductions in inflammation and senescence-associated markers. Microbial changes observed in humans are shown in purple, whereas alterations identified in mice are shown in purple. Created in BioRender. Jamous, J. (2026) https://BioRender.com/cgs86ep.

Quercetin is a dietary flavonoid abundant in fruits and vegetables that has gained considerable interest in geroscience due to its senolytic properties [147,148]. Its molecular structure enables it to directly scavenge reactive oxygen and nitrogen species, and activate Nrf2, a key transcription factor that induces antioxidant and cytoprotective genes (Figure 5) [148]. Quercetin has been shown to reduce senescent fibroblast numbers by selectively suppressing autophagic activity, thereby increasing endoplasmic reticulum stress and promoting apoptosis. It also decreases NF-κB p65 nuclear translocation and downregulates expression of key SASP genes, including IL-6, MMP3, VEGF, and CXCL12, among others. In addition, quercetin targets anti-apoptotic BCL-2 family proteins, particularly BCL-xL and BCL-2, thereby reducing the apoptotic resistance characteristic of senescent cells [149]. Through inhibition of PI3K/Akt/mTOR and STAT3 signaling, quercetin induces apoptosis in non-small-cell lung cancer and primary effusion lymphoma cells [150,151]. Taken together, these findings demonstrate that quercetin not only eliminates senescent cells, but also reduces SASP production, contributing to a healthier cellular environment and potentially delaying age-related diseases. On the other hand, dasatinib is a broad-spectrum tyrosine kinase inhibitor that targets multiple pro-survival kinases upregulated in senescent cells, including SRC/SFKs, BCR-ABL, and the EPHA2 receptor, consequently disrupting their survival signaling and making senescent cells more susceptible to apoptosis [152]. Overall, quercetin has been shown to reduce senescence in endothelial cells, adipocytes, and fibroblasts [149,153,154,155], whereas dasatinib is primarily effective in preadipocytes [154]. To broaden the range of senescent cell types targeted, researchers introduced the combination therapy of dasatinib and quercetin (D+Q), which act synergistically to selectively eliminate senescent cells in both animal models and humans [63,137,142,156,157]. In mice, treatment with D+Q has been shown to eliminate senescent oligodendrocyte progenitor cells and reduce Ab plaque-associated inflammation and cognitive decline [156], decrease chondrocyte senescence and alleviate facet joint degeneration [157], and mitigate renal damage [145]. Moreover, ongoing clinical trials are evaluating the feasibility, safety, and tolerability of D+Q therapies for modulating AD progression, for bone loss in postmenopausal women, and to improve physical function in patients with idiopathic pulmonary fibrosis [143,144,158].

#### 5.3.2. Impact of Senolytics on the Gut Microbiota

Emerging evidence suggests that senolytics, particularly D+Q, can influence the gut microbiota, indicating that their effects extend beyond clearing senescent cells (Figure 5) [159,160]. In aged mice, D+Q administration shows notable improvements in intestinal health, including reduced inflammation, attenuation of cellular senescence, and shifts in microbial composition [159]. In aged mice, it increases beneficial bacteria such as *Akkermansia* and decreases inflammation-associated taxa, including *Proteobacteria*, *Staphylococcus*, *Clostridium cocleatum*, and *Roseburia* [159]. Similar patterns have been observed in Crohn’s disease patients, where D+Q treatment elevates anti-inflammatory and health-promoting bacteria, including *Bacteroides* and members of the phylum *Actinobacteria*, particularly the genus *Bifidobacterium* and the family *Eggerthellaceae*, while reducing potentially pathogenic taxa such as *Streptococcus*, *Megamonas*, and *Sutterellaceae* [161].

D+Q also enhances the physical structure and function of aged intestines by promoting the regeneration of villi and crypts [162], both essential for nutrient absorption [163]. Moreover, it supports progenitor cell proliferation while restoring nitric oxide levels, which helps maintain gut barrier integrity and function [162]. These intestinal effects align with findings in other tissues, such as the intervertebral disc, where sustained D+Q treatment preserves structural integrity and reduces senescence markers [164]. Together, these findings suggest that D+Q not only mitigates cellular aging but also improves gut health by supporting immune function, metabolic balance, and gut barrier integrity [154,159,160,165]. Although this field of research is still relatively novel and limited, current evidence positions D+Q as a senolytic intervention capable of modulating microbiota composition and reducing intestinal inflammation in aging.

### 5.4. GLP-1R Agonists

Glucagon-like peptide-1 (GLP-1) is an incretin hormone that is released from the gut in response to food consumption. It plays a crucial role in glucose homeostasis by promoting insulin secretion, inhibiting glucagon release, delaying gastric emptying, and inducing satiety. GLP-1 binds to its receptors (GLP-1R) located in the intestine, pancreas, heart, brain, lung, and kidney [56,166,167]. However, the natural form of GLP-1 becomes rapidly degraded by the DPP-4 enzyme in the bloodstream [168]. To overcome this limitation, GLP-1R agonists have been developed as synthetic drugs to mimic the effects of natural GLP-1 while showing a higher resistance to DPP-4 degradation, providing a longer therapeutic effect for managing blood sugar levels and improve metabolic function. Current GLP-1R agonists approved by FDA to treat T2D include semaglutide, dulaglutide, albiglutide, exenatide, liraglutide, lixisenatide, and tirzepatide. In addition, liraglutide, semaglutide, and tirzepatide have been approved for chronic weight management in individuals with obesity. Although GLP-1R agonists were originally developed to treat T2D and are thought to influence aging by mitigating metabolic syndrome [56], the widespread distribution of GLP-1R throughout the body suggests a broader therapeutic potential. In fact, in 2024, semaglutide was FDA-approved to reduce the risk of major adverse cardiovascular events, including cardiovascular death, heart attack, and stroke in patients with obesity or diabetes. Recent evidence indicates shared mechanistic pathways between neurodegenerative diseases and T2D [169], highlighting the potential of GLP-1R agonists as therapeutic agents to ameliorate neurodegeneration. Furthermore, GLP-1 levels in the human gut decline with age [170], a finding that may connect reduced GLP-1 signaling to the onset of age-related diseases.

#### 5.4.1. Mechanisms of Action of GLP-1R Agonists

**Figure 6:**
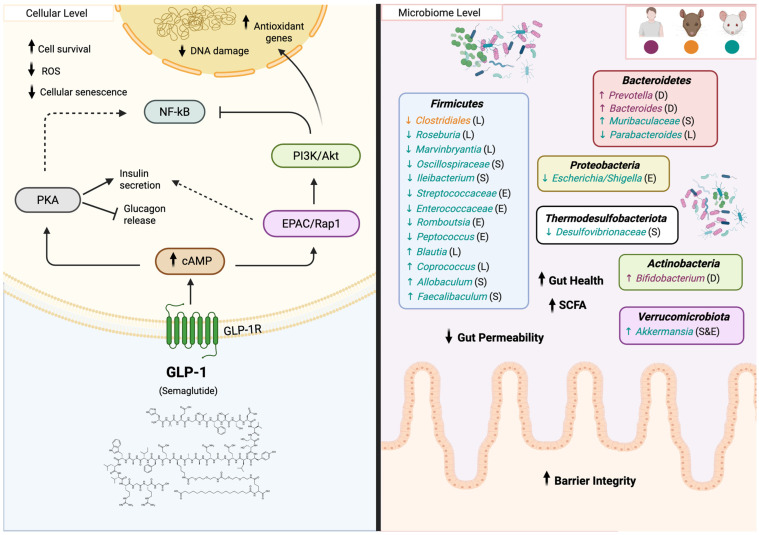
Overview of the pleiotropic effects of GLP-1R agonists at the cellular and microbiome levels. At the cellular level, GLP-1R activation (e.g., semaglutide) increases cAMP signaling, engaging PKA and EPAC/Rap1 pathways to modulate PI3K–Akt and NF-κB signaling. Purple indicates gut microbiome changes in humans, orange in rats, and blue in mice. GLP-1R agonists remodel gut microbial composition in mice and humans, with distinct microbial responses associated with individual agonists—liraglutide (L), semaglutide (S), dulaglutide (D), and exenatide (E)—supporting SCFA production, reduced gut permeability, improved barrier integrity, and overall gut health. Created in BioRender. Garzon-Escamilla, N. (2026) https://BioRender.com/1q0oyqb.

Binding GLP-1R agonists to GLP-1R increases intracellular cAMP, which activates protein kinase A (PKA) (Figure 6). In pancreatic b-cells, PKA enhances insulin secretion, whereas in pancreatic a-cells it inhibits glucagon release, together contributing to lower blood glucose levels. cAMP can also activate EPACs (Exchange Proteins directly Activated by cAMP), which in turn activate Rap1, further supporting insulin secretion. In addition, GLP-1 activates the PI3K/Akt pathway, a key signaling cascade that promotes pancreatic b-cells survival and function [56]. Through these pathways, GLP-1R agonists protect cells from oxidative stress by boosting antioxidant defenses, preventing DNA damage, and suppressing premature senescence [171,172]. Moreover, GLP-1R agonists are able to reduce food intake by activating GLP-1R-expressing neurons in the hypothalamus [173], playing an important role in regulating energy homeostasis. Also, GLP-1 binding to intestinal receptors can cause intestinal muscles to relax and reduce stomach motility, slowing down the process of gastric emptying [174,175]. On the other hand, research in animal models and human preclinical studies have shown that GLP-1R agonists exert protective effects against neurodegenerative diseases such as Parkinson’s and Alzheimer’s disease [56,176]. GLP-1R agonists reduce pro-inflammatory cytokines by suppressing the NF-κB pathway, improve mitochondrial function and protein homeostasis, and regulate autophagy through cAMP/PKA and PI3K/Akt signaling. [56,176]. Similarly, GLP-1R agonists provide cardioprotection by reducing oxidative stress and improving cardiomyocyte survival and endothelial function [177,178]. Together, these findings demonstrate a broad array of beneficial effects of GLP-1R agonists that extend far beyond metabolic regulation, positioning these drugs as a promising therapeutic approach to promote healthspan and ameliorate age-related diseases. To improve metabolic outcomes, current research is also exploring the potential of multi-receptor agonists, such as tirzepatide, the first FDA-approved GIP/GLP-1 dual receptor agonists [179,180,181], and retatrutide, a novel triple agonist drug targeting GLP-1, GIP, and glucagon receptors [182,183]. However, more research is needed to determine whether multi-receptor agonists could have a greater impact on slowing the aging process than mono-receptor agonists. Next, we will examine emerging evidence that GLP-1 signaling can influence the gut microbiota, highlighting an additional pathway through which these drugs could impact systemic health and aging.

#### 5.4.2. Impact of GLP-1R Agonists on the Gut Microbiota

GLP-1R agonists have been shown to influence gut microbial diversity in vivo, with several studies demonstrating modulation of both microbial richness and community composition in animal and human models (Figure 6) [166,167,184,185,186]. Liraglutide has been shown to change the microbial composition in both obese and diabetic rats by increasing *Bacteroidetes* and reducing the abundance of *Firmicutes*, particularly within families such as *Lachnospiraceae* and *Clostridiales* [184]. This modulation appears to enhance the *Bacteroidetes*-to-*Firmicutes* ratio, which is favorable for weight management. Furthermore, in a mouse model of metabolic disease, liraglutide decreases genera previously associated with obesity, such as *Roseburia*, *Marvinbryantia* and *Parabacteroides*, and enriches lean-associated genera like *Blautia* and *Coprococcus* [187]. Most of these bacteria belong to the *Lachnospiraceae* family, and the seemingly inconsistent shifts highlight that, as with many compounds, liraglutide’s effects on the gut microbiota are highly context dependent. Specific bacterial changes vary according to the host’s disease state or model organism studied, emphasizing the importance of considering host context when interpreting microbiome shifts and limiting the ability to generalize findings. On the other hand, semaglutide consistently restored gut microbial diversity and composition across high-fat diet (HFD) and diabetic mouse models, increasing beneficial bacteria such as *Akkermansia*, *Muribaculaceae*, *Allobaculum*, and *Faecalibaculum*, while reducing dysbiosis-associated taxa, including *Desulfovibrionaceae*, *Oscillospiraceae*, and *Ileibacterium* [188,189,190]. These changes were associated with improved gut barrier integrity, reduced intestinal permeability, enhanced SCFA production, and anti-inflammatory effects. Shifts in *Dubosiella*, *Lactobacillus*, and *Lachnospiraceae* appear to be context-dependent and remain somewhat controversial.

Fewer studies have investigated the microbiome effects of dulaglutide and exenatide. One study reported that long-term dulaglutide treatment in patients with T2D increased the abundance of *Bacteroides*, *Prevotella*, and *Bifidobacterium* [191]. In diabetic mouse models, exenatide reduced conditionally pathogenic families such as *Streptococcaceae* and *Enterococcaceae* [192] and increased the abundance of *Akkermansia*. At a genus level, it decreased the abundance of *Romboutsia*, *Peptococcus*, and *Escherichia/Shigella* [192].

Although further research on different GLP-1R agonists is needed to fully assess the global microbiome effects of these potential anti-aging therapies, these findings indicate that these agents consistently shift the gut microbiota toward a metabolically favorable profile. However, the specific bacterial taxa affected are highly context-dependent, varying with host metabolic status, disease model, and experimental conditions.

### 5.5. Spermidine

Spermidine, a natural polyamine found in living organisms and a variety of foods, has gained recognition as a strong anti-aging compound over the past years [12,60]. It is considered as a promising agent to combat age-related diseases such as neurodegenerative diseases, metabolic disorders, cardiovascular conditions, and cancer due to its crucial role in a wide range of essential cellular processes such as DNA stability, lipid metabolism, autophagy, cell growth, and apoptosis among many others [59,60]. According to the FDA, spermidine is not currently recognized as a drug, but a dietary supplement considered as Generally Recognized as Safe (GRAS). A key factor behind spermidine’s ability to influence such a broad array of biological functions is the ability it has to directly interact with negatively charged molecules including DNA, RNA, ATP, proteins, and lipids [60,193]. Spermidine, found in many tissues and organs across the human body, has been shown to decline significantly with age, specifically in people over the age of 60 [194,195]. Interestingly, this age coincides with the onset of most age-related diseases, implying a potential relationship with spermidine loss. On the other side, centenarians and nonagenarians often exhibit higher levels of spermidine [194]. In addition, numerous studies demonstrate that spermidine supplementation can mitigate age-related changes and extend lifespan across a range of model organisms, including mice, yeast, worms, flies, and cultured mammalian cells [60,195,196,197,198]. Overall, these findings suggest a strong connection between spermidine and longevity.

#### 5.5.1. Mechanisms of Action of Spermidine

**Figure 7:**
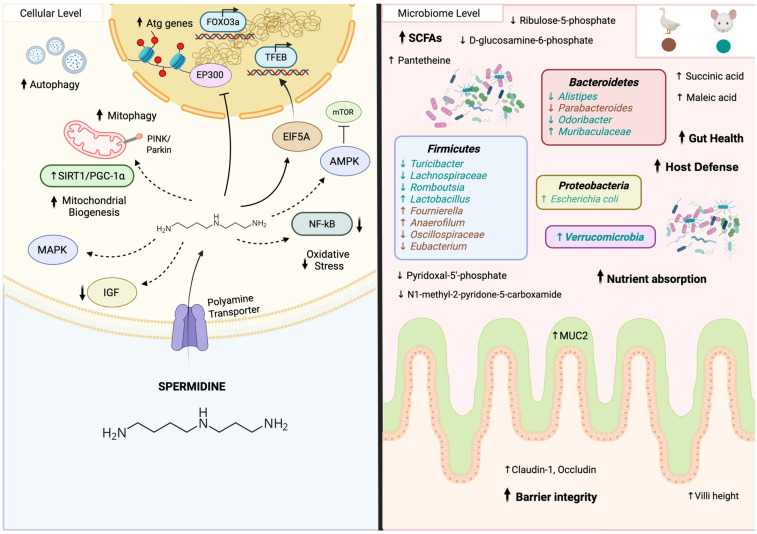
Overview of the pleiotropic effects of spermidine at the cellular and microbiome levels. Spermidine activates autophagy and longevity-associated pathways, including AMPK activation, mTOR inhibition, FOXO3a and TFEB activation, epigenetic regulation via EP300, mitophagy (PINK/Parkin), mitochondrial biogenesis (SIRT1/PGC-1a), and oxidative stress resistance, while suppressing NF-κB-mediated inflammation. At the microbiome level, studies in mouse (blue) and goose (brown) models have shown that spermidine improves gut barrier integrity and nutrient absorption, increased SCFA production and metabolite remodeling, alongside compositional shifts favoring beneficial taxa and reduced potential detrimental bacteria. Created in BioRender. Medina-Cardena, M. (2026) https://BioRender.com/ubyn0im.

Spermidine displays a diverse array of mechanisms to promote beneficial effects on the host and has demonstrated a remarkable ability to influence most hallmarks of aging [199]. This polyamine compound interacts with essential intracellular mechanisms and pathways, encompassing DNA stability and homeostasis, gene regulation, apoptosis, and oxidative stress (Figure 7) [59,200,201]. The most-studied mechanism of action of spermidine is through the regulation of autophagy [60,202]. Spermidine increases autophagy by upregulating the Atg genes, and enhances the synthesis of TFEB, a master regulator of autophagy [60,199]. In addition, this compound inhibits the acetylation of p300 (EP300), an acetyl transferase that directly stimulates the acetylation of different autophagy essential proteins [59,60,203]. Moreover, spermidine has shown to activate the PINK1-PDR1-dependent mitophagy pathway in *C. elegans* [197], and it promotes mitochondrial bioenergetics in human-induced pluripotent stem cells [201]. This autophagy-enhancing compound has proven to have therapeutic benefits in neurodegenerative diseases such as Alzheimer’s and Parkinson’s disease by ameliorating age-associated memory decline and dementia [60,197,204,205,206,207]. Additionally, impaired metabolism of spermidine is frequently observed in cancer [59]. The autophagic effects induced by spermidine have shown to reduce tumor growth and favor immunosurveillance [59,208,209]. Previous studies have provided evidence that supplementation with this anti-aging compound significantly reduces inflammation by suppressing NF-κB signaling and the production of pro-inflammatory cytokines and reactive oxygen species, while simultaneously promoting the production of anti-inflammatory cytokines [60,210]. Lipid metabolism regulation is considered another of the multiple modes of action of spermidine [60]. Spermidine downregulates expression of lipogenic genes through the AMPK signaling pathway [211], suppresses necrotic core formation and lipid accumulation by stimulating cholesterol outflow [212], and promotes the differentiation and maturation of adipocytes [213]. Even though the precise mechanisms remain unknown, spermidine supplementation preserves telomere length in mice and is capable of suppressing the induction of senescence both in vitro and in vivo [214]. Ultimately, spermidine has shown effects in multiple signaling pathways. It promotes the expression of SIRT1/PGC-1α in mitochondrial biogenesis, enhances FOXO3a activity, and upregulates the expression of MAPK genes, while suppressing IGF signaling and inhibiting mTOR [60,199]. In conclusion, the evidence presented highlights the diverse mechanisms of action of spermidine, demonstrating its impact on various pathways beyond autophagy. These mechanisms work synergistically to boost spermidine’s anti-aging effects, promoting health, and extending longevity.

#### 5.5.2. Impact of Spermidine on the Gut Microbiota

Emerging evidence suggests that spermidine’s impact on health involves modifications to the gut microbiome (Figure 7) [215]. Recent studies in mice have revealed that this polyamine compound significantly influences the composition and diversity of gut microbiota and prevents intestinal dysbiosis in mice [215,216]. Spermidine treatment led to notable shifts in microbial populations, including a reduction in the abundance of potentially harmful bacteria such as *Turicibacter*, *Alistipes*, and *Romboutsia*, while promoting an increase in beneficial species such as *Lactobacillus* [215]. Additionally, mice treated with high levels of spermidine exhibited elevated levels of the phylum *Verrucomicrobia*, the family *Muribaculacea*, and the species *Escherichia coli*, whereas taxa often associated with dysbiosis, including the family *Lachnospiraceae* and the genus *Odoribacter* were reduced [215,216]. However, spermidine not only affects bacterial populations in the intestine, but also modulates gut metabolites, increasing metabolites such as succinic acid, maleic acid, and pantetheine, while decreasing metabolites like N1-methyl-2-pyridone-5-carboxamide, pyridoxal 5′-phosphate, ribulose 5-phosphate, and D-glucosamine 6-phosphate among others [215]. Even though spermidine clearly induces shifts in gut metabolites, further research is needed to uncover the biological significance and potential impact of these metabolites on host health. After administering spermidine to diet-induced obese mice, they showed an increase in butyrate and other SCFAs in their intestines, which are known to support epithelial barrier integrity and reduce endotoxemia [217]. Additionally, spermidine improved the gut barrier function by strengthening tight junctions and enhancing the mucosal barrier due to an upregulation of mucin, claudin 1, and occludin [217]. Spermidine also exerted protective effects on the gut itself. Its administration in mice prevented colitis development in a dose-dependent and PTPN2-dependent manner in both intestinal epithelial cells and monocytic myeloid cells [216]. Beyond the gut structure and microbiota, spermidine promoted the differentiation of M2-like anti-inflammatory macrophages, contributing to the resolution of intestinal inflammation in mice [216]. In geese, spermidine showed a significant increase in the abundance of *Fournierella* and *Anaerofilum*, while decreasing the abundance of the family *Oscillospiraceae*, *Parabacteroides*, and inhibiting *Eubacterium* colonization [218]. Overall, spermidine modulates the gut flora by promoting the colonization of beneficial bacteria in geese gut [218]. Furthermore, spermidine treatment improves gut structural integrity by increasing villi heights in the jejunum and ileum, contributing to an increase in nutrient absorption and overall gut function in mice and geese [215,218]. This supplement has been shown to drive modifications in the microbiota, including shifts in microbial abundance and composition, while also enhancing gut integrity. In order to better comprehend the effects of spermidine on the gut microbiome, further research is needed to clarify the specific alterations in gut flora and their broader implications on health, including research on germ-free animal models to determine whether spermidine interacts with the microbiome as a mechanism of action, or if the microbial shifts observed in the gut are simply a consequence of supplement intake. Finally, studies directly correlating these effects to humans are essential to fully elucidate the potential therapeutic applications of spermidine.

### 5.6. Sirtuin Activator Compounds

Silent information regulator 2 (Sir2) proteins, sirtuins, are a family of NAD-dependent histone deacetylases highly conserved evolutionarily [62,219,220]. The sir2 gene was initially identified as a key regulator of yeast replicative longevity [221]. Since then, sir2 homologs have been recognized across diverse species through similarity in the conserved central catalytic core domain [222]. Mammals possess seven sirtuins, identified as SIRT1-SIRT7 [220,223]. Among them, SIRT1, SIRT3, and SIRT6 are naturally activated by caloric restriction (CR) and physical activity, and their elevated expression has been associated with biological functions such as DNA repair, genome integrity, anti-inflammatory responses, stress resistance, metabolic health, mitochondria biogenesis and the cell cycle [220,224]. Moreover, research highlights a link between sirtuin overexpression and lifespan extension in yeast, *Drosophila*, *C. elegans*, and mice [219,220,224,225,226]. Sirtuins have been associated not only with lifespan extension but also with healthspan extension, due to their roles in modulating multiple age-related diseases including cardiovascular conditions [227,228], neurodegeneration [229,230,231], cancer [232,233,234], and metabolic disorders [235,236,237]. Given the significant health advantages observed, identifying sirtuin activating compounds (STACs) attracted significant attention in the aging field. On one hand, natural sirtuin activators include plant-derived polyphenols such as resveratrol, curcumin, and pterostilbene that have been shown to extend lifespan in various model organisms, but their effects in humans appear weaker, and research is still ongoing [238,239,240,241,242]. On the other hand, synthetic activators such as SRT1720, SRT2104, and MDL-800 are designed to provide stronger and more selective activation of sirtuin pathways, but questions about their safety in humans continue to remain unresolved [243,244,245]. Finally, NAD^+^ precursors like nicotinamide riboside (NR) and nicotinamide mononucleotide (NMN) support sirtuin function indirectly by increasing cellular NAD^+^ availability, the essential cofactor required for sirtuin activity [246,247]. Although sirtuins have long been thought to be involved in lifespan extension, the use of their modulators as direct anti-aging compounds remains highly debated. Reported lifespan benefits have been inconsistent and difficult to reproduce, especially in mammals [248,249,250]. There are also questions about whether STACs act directly on sirtuins or influence them indirectly as part of broader cellular effects, and many of these substances have limited bioavailability, breaking down too quickly or being poorly absorbed to reach effective levels in the body [251,252]. Even though some questions still need to be resolved through research, sirtuins remain scientifically interesting and biologically relevant in longevity research due to their connection to key cellular processes linked to healthspan.

#### 5.6.1. Mechanism of Action of STACs

**Figure 8:**
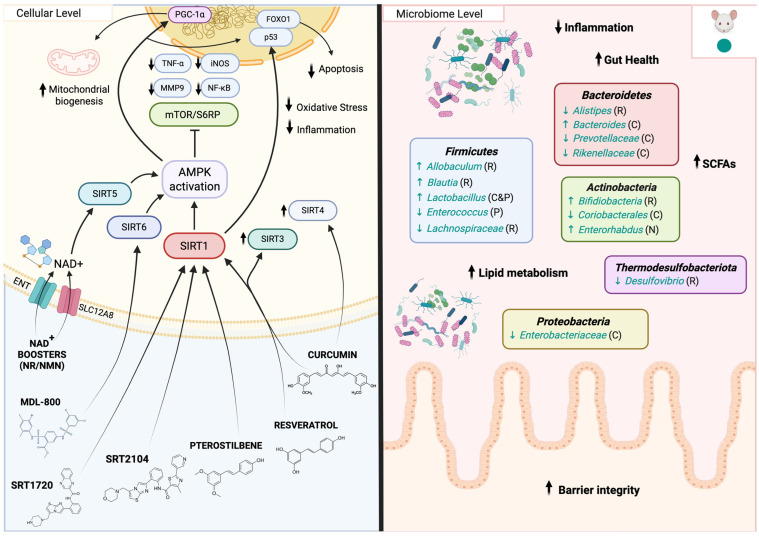
Overview of the pleiotropic effects of STACs at the cellular and microbiome levels. At the cellular level, STACs promote cellular stress resistance and metabolic homeostasis via SIRT1-AMPK activation, suppression of mTOR/NF-κB signaling, reduced oxidative stress, apoptosis, and inflammation, and enhanced mitochondrial biogenesis. These mechanisms reshape the gut microbiome by reducing dysbiosis, increasing SCFA-producing and beneficial bacteria while limiting pathobionts. Gut microbiome alterations induced by STACs are shown in blue for mice, with microbial shifts associated with specific compounds, including resveratrol (R), curcumin (C), pterostilbene (P), and NAD^+^ boosting compounds (N), highlighting drug-dependent effects on lipid metabolism, barrier integrity, and overall gut health. Created in BioRender. Trent, J. (2026) https://BioRender.com/sdq7gk7.

Sirtuins are key nutrient-sensing proteins that contribute to the beneficial effects of CR, the most well-established intervention known to extend lifespan in model organisms [246]. Advances in this area of research have led to the identification of numerous small molecules, including natural products and synthetic compounds, which have emerged as STACs capable of improving aging-associated phenotypes and diseases. Resveratrol, curcumin, and pterostilbene are some of the most common natural STACs [253,254,255]. These compounds exert beneficial effects by improving oxidative stress, decreasing inflammation, inhibiting telomere shortening, and decreasing cellular senescence (Figure 8) [256,257]. While these natural STACs increase lifespan in yeast, worms, fish, and fruit flies [224,226,258,259], studies in mammals indicate benefits primarily linked to healthspan and stress resistance rather than consistent lifespan extension [238,239,240,241,242,260]. Resveratrol enhances the expression of antioxidant genes and neuroprotective factors by SIRT1 and SIRT3 activation in immortalized lymphocytes from Alzheimer’s disease patients [261], and improves neuronal flexibility in the hippocampal region by SIRT1 activation [262]. Moreover, low concentrations of resveratrol stimulate SIRT1, resulting in the deacetylation of liver kinase B1, which serves as an upstream kinase for AMPK, consequently activating it [246]. In addition, resveratrol attenuates TNF-α–induced inflammatory signaling in fibroblasts, in part through activation of SIRT1. This is associated with reduced expression of pro-inflammatory cytokines, inducible nitric oxide synthase (iNOS), and matrix metalloproteinase-9 (MMP-9), along with decreased acetylation and transcriptional activity of NF-κB and suppression of mTOR/S6 ribosomal protein (S6RP) signaling, collectively leading to diminished inflammatory mediator production [263]. Silencing SIRT1 eliminates these anti-inflammatory effects, indicating that resveratrol’s action is SIRT1-dependent [263]. Curcumin has been found to play beneficial roles in myocardial infarction and neurological disorders by activation of SIRT1 [264,265,266]. Moreover, curcumin treatment increases the activation levels of AMPK in rat and mice muscles [267,268]. This natural compound also elevates the level of SIRT3 and SIRT4 in mouse models and reduces oxidative stress and inflammatory markers, having a protective role against age-related disorders [269,270]. Similarly, pterostilbene also plays protective roles in aging by decreasing inflammatory markers and oxidative stress [271]. In obese mice models, pterostilbene promotes thermogenesis and mitochondrial biogenesis by stimulating the SIRT1/PGC-1α/SIRT3 pathway. It also stimulates the SIRT1-FOXO1/p53 signaling pathway and reduces the apoptosis rate in skeletal muscle cells caused by ischemia–reperfusion injury [246]. Synthetic STACs were developed to mimic natural polyphenols and generally have more potent effects in vitro [272]. SRT1720 is a first generation SIRT1 activator that has been found to increase lifespan and healthspan in mice by supporting metabolic health, reducing inflammation and oxidative stress, improving cardiovascular function, liver function, and physical performance [273,274,275]. Also, SRT1720 prevents senescence in vascular smooth muscle cells by activating the SIRT1/p-AMPK pathway, resulting in decreased expression of p53, p21 and p16 proteins and restoring telomere length [276]. SRT2104, part of the second generation of SIRT1 activators, has shown impressive stability in the mitochondria and plasma of both human and mouse livers [277]. SRT2104 activates SIRT1, leading to p53 deacetylation, which reduces pro-apoptotic signaling, and also leading to NF-κB deacetylation, suppressing pro-inflammatory cytokine production and NLRP3 inflammasome activation, contributing to cellular protection in disease models [256,278]. In addition, in a mouse model of induced acute inflammation, SRT2104 demonstrated greater in vivo anti-inflammatory effectiveness than comparable compounds within the same activation range [277]. Finally, the STAC MDL-800 stimulates SIRT6 through binding to an allosteric site, facilitating the deacetylation of histones H3K9 and H3K56 in human hepatocellular carcinoma cells [279]. MDL-800 mitigates the age-related reduction in DNA repair in mouse-derived cell lines. Nonetheless, additional studies have uncovered off-target effects from these natural and synthetic activators, suggesting that its beneficial effects may not be solely mediated by sirtuins [243,257,273,280,281,282,283,284]. Additionally, a new category of sirtuin activators known as NAD^+^ boosting molecules is becoming popular as a method to replenish NAD^+^ levels in older adults, enhancing the activity of multiple NAD^+^-dependent enzymes, including sirtuins, in a single intervention. There are two methods to modulate NAD^+^ levels, either by directly restoring NAD^+^ with precursor supplements, or by overexpressing enzymes involved in NAD^+^ biosynthesis such as NAMPT and NMNAT, thereby enhancing NAD^+^ synthesis. Supplements containing NAD^+^ precursors are NMN and NR [246]. Long-term NAD^+^ booster supplementation alleviates the decline in physiological age-related functions in mice by enhancing energy metabolism [285]. NAD^+^ boosting molecules have partially reversed the loss of skeletal muscle, and it also safeguards against β-amyloid oligomer-induced cognitive decline and neuronal cell death in a mouse model of Alzheimer’s [286]. In addition, certain NAD^+^ boosting compounds have been identified as cell-permeable activators of SIRT5 [287]. In general, sirtuin activators have anti-aging effects by directly or indirectly boosting sirtuin activity to regulate nutrient sensing, mitochondrial biogenesis, redox stability, and stress-response pathways—mainly through SIRT1, SIRT3, SIRT6 and AMPK-dependent signaling—thereby lessening inflammation, oxidative damage, apoptosis, and metabolic issues while enhancing cellular resilience and longevity.

#### 5.6.2. Impact of STACs on Gut Microbiome 

Recent studies have found that there is a bidirectional relationship between sirtuins and gut microbiota [288]. Sirtuins have been found to play an essential role in maintaining the composition of the intestinal microbiome by maintaining the intestinal barrier and assisting the mucosal immune mechanism in mouse models (Figure 8) [289]. SIRT3-deficient mice exhibited increased gut inflammation and tumors, displaying fewer beneficial bacteria such as *Lactobacillus reuteri* and *Lactobacillus taiwanensis*, and a higher presence of pathobionts, including *Escherichia* and *Shigella* [289]. On the other hand, research shows that the gut microbiota can also impact the activity of sirtuins by affecting essential transcriptional co-activators, transcription factors, and enzymatic pathways related to mitochondrial biogenesis, including proteins like PGC-1α, SIRT1, and AMPK [290,291]. Additionally, metabolites generated by gut microbiota through the fermentation of indigestible food components can influence the roles of sirtuins in reducing oxidative stress and inflammation in mice [292]. Also, the gut microbiota engages with STACs to impact molecular pathways that combat aging and inflammation, while promoting certain gut microbiota populations to boost immune function [293]. Resveratrol improves the gut barrier function and decreases pathogenic bacteria under obesogenic conditions such as *Desulfovibrio*, *Alistipes*, and certain members of the *Lachnospiraceae* family, while enriching SCFA-producing bacteria such as *Allobaculum*, *Bacteroides*, and *Blautia* [294]. Other STACs like curcumin and pterostilbene also show protective effects against gut inflammation and change gut microbiome composition [288]. Oral curcumin administration significantly alters the gut microbiota in mice, promoting beneficial taxa such as *Bifidobacteria*, *Lactobacilli*, and butyrate-producing bacteria, while reducing the relative abundance of *Prevotellaceae*, *Coriobacterales*, *Enterobacteriaceae*, and *Rikenellaceae*, which have been associated with inflammation and systemic disease [295,296,297]. Moreover, pterostilbene decreases the presence of opportunistic bacteria, like *Enterococcus*, while enhancing beneficial bacteria such as *Lactobacillus*, thus altering the gut microbiota in a murine model [298]. Lastly, NAD^+^ boosting molecules increase the abundance of *Actinobatceria* and *Enterorhabdus* and enhance lipid metabolism [299]. In summary, these investigations collectively emphasize sirtuin activators as essential regulators of the bidirectional sirtuin–gut microbiota interaction, showing that substances like resveratrol, curcumin, pterostilbene, and NAD^+^ precursors not only boost sirtuin function to maintain intestinal barrier integrity, lessen inflammation, and foster mitochondrial and metabolic wellness, but also alter the gut microbiota to a favorable, SCFA-generating profile, highlighting their therapeutic promise in advancing gut homeostasis and promoting healthy aging.

### 5.7. SGLT2 Inhibitors

Sodium-glucose co-transporter 2 (SGLT2) is a protein primarily found in the kidneys responsible for reabsorbing glucose back into the bloodstream [300,301]. SGLT2 inhibitors are a class of antidiabetic drugs that increase urinary glucose excretion, thus lowering blood glucose levels [300]. As of now, there are four FDA-approved SGLT2 inhibitors: canagliflozin, dapagliflozin, empagliflozin, and ertugliflozin [302]. Unlike other antidiabetic medications, SGLT2 inhibitors function independently of insulin production, making them an effective strategy against insulin resistance [303,304,305]. Furthermore, these drugs have demonstrated to reduce cellular senescence, pro-inflammatory cytokines production and oxidative stress, promote autophagy, regulate key nutrient-sensing pathways, and modulate gut microbiota [301,306,307,308,309,310,311]. Consequently, SGLT2 inhibitors have shown important benefits in the management of common age-related conditions including cardiovascular disease, chronic kidney disease, cancer, neurodegenerative diseases and non-alcoholic fatty liver disease [306,308,312,313,314,315,316,317,318,319]. Although SGLT2 inhibitors were initially developed for managing T2D, these findings highlight the importance of better understanding their mechanism to support potential drug repurposing.

#### 5.7.1. Mechanism of Action of SGLT2 Inhibitors

**Figure 9:**
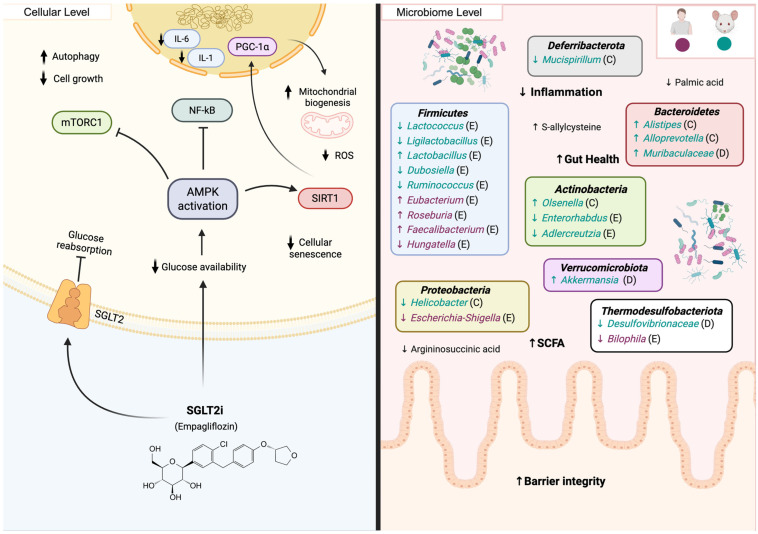
Overview of the pleiotropic effects of SGLT2 inhibitors at the cellular and microbiome levels. SGLT2 inhibitors reduce glucose reabsorption and availability, activating AMPK and suppressing mTORC1 and NF-κB signaling, with downstream activation of SIRT1 and PGC-1α. SGLT2 inhibitors remodel gut microbial composition in a drug-specific manner, with microbial shifts linked to canagliflozin (C), dapagliflozin (D), and empagliflozin (E), promoting SCFA-producing bacteria, reducing gut permeability and inflammation, and improving barrier integrity and overall gut health. Microbial changes observed in humans are shown in purple, while those observed in mice are shown in blue. Created in BioRender. Garzon-Escamilla, N. (2026) https://BioRender.com/zyurrw5.

SGLT2 inhibitors require direct binding to SGLT2 to exert their renal glucose-lowering effects; however, their cellular entry and many downstream signaling actions occur independently of SGLT2 [320,321]. Recent evidence indicates that a substantial portion of the pleiotropic beneficial effects of SGLT2 inhibitors are mediated through their ability to attenuate cellular senescence, largely by activating AMPK, increasing SIRT1 activity, decreasing ROS levels, and suppressing NF-κB signaling and mTORC1 activity (Figure 9) [55,309,310,311,322,323,324]. Canagliflozin has been shown to increase the lifespan of male mice by lowering the peak blood glucose levels after food intake [318], as well as regulating key nutrient-sensing pathways associated with healthspan such as mTOR, AMPK, and FGF21 [307]. These findings highlight the potential of canagliflozin as a sex-specific therapeutic agent for aging-related diseases, particularly in males. Empagliflozin was found to exert neuroprotective effects in zebrafish models of Parkinson’s disease by enhancing ketogenesis and autophagy, mimicking the therapeutic benefits of the ketogenic diet [15]. It has been demonstrated that the ketogenic diet induces alterations in the gut microbiota, metabolites, and both systemic and intestinal inflammation [15,16], suggesting that at least some of the neuroprotective effects of the ketogenic diet are mediated by changes in the gut microbiota. Additionally, it has been reported that empagliflozin mitigates neuroinflammation in high-fat diet-fed mice by reducing astrocyte activation and promoting autophagy by modulating the Akt-mTOR pathway, and improving the gut microbiota profile [308]. As a result, this SGLT2 inhibitor could potentially improve the gut–brain axis and contribute to neuroprotective and anti-aging effects. Dapagliflozin improves cardiovascular health by reducing myocardial hypertrophy and fibrosis through SIRT1 expression in mice cardiomyocytes [325]. Furthermore, it reduces the risk of worsening heart failure and cardiovascular death in humans [314]. On the other hand, by suppressing TLR-4 and NF-κB, this drug reduces the secretion of pro-inflammatory mediators such as IL-1 and IL-6 and promotes the shift from pro-inflammatory M1 to anti-inflammatory M2 macrophages [324,326]. In addition, it also reduces oxidative damage, supporting redox balance and protecting mitochondrial function [309,327,328]. In mouse models, dapagliflozin reduces oxidative stress by boosting antioxidant activity and by upregulating SIRT1 and PGC-1α expression, improving insulin signaling and brain mitochondrial function [311,329,330,331]. Moreover, it improves learning and memory in mice, and prevents cognitive decline and neuronal dysfunction in rats [329,332,333,334]. At the cellular level, dapagliflozin has demonstrated anti-senescence effects by reducing SASP factors through AMPK and SIRT1 activation [335]. In high-glucose conditions, it suppresses inflammatory cytokines and enhances autophagy via AKT/mTOR inhibition [335]. In the long-term, it reduces renal senescence markers and SASP factors [336]. Finally, collagen production naturally declines with age, but dapagliflozin preserves skin collagen in mice by inhibiting mast cell activation and MMP-1 secretion [337]. Finally, while ertugliflozin is effective for managing diabetes, it has shown fewer benefits related to aging as compared to the previously discussed SGLT2 inhibitors in humans. Although clinical trials have confirmed its cardiovascular safety, it did not show significant results in the reduction in major cardiovascular events such as heart failure [338]. Nonetheless, a previous clinical trial has associated ertugliflozin with body-weight loss [338]. Given the limited data on this drug’s effects beyond diabetes, further research is needed to assess its potential in other conditions and to uncover the molecular mechanisms that may support healthy aging.

#### 5.7.2. Impact of SGLT2 Inhibitors on Gut Microbiota

Increasing evidence demonstrates that SGLT2 inhibitors modulate the gut microbiome in ways that may impact the development of age-related diseases (Figure 9). For example, canagliflozin administration increases the relative abundance of *Olsenella*, *Alistipes*, and *Alloprevotella* in diabetic mice with cardiovascular disease [339], taxa that have been associated with improved metabolic and anti-inflammatory profiles [340]. It also reduced the abundance of *Helicobacter* and *Mucispirillum* [339], two genera commonly linked to gut inflammation and impaired barrier function [341,342], with the role of *Mucispirillum* considered more context-dependent. Beyond suppressing astrocyte activation, empagliflozin ameliorates high-fat diet-induced neuroinflammation by decreasing the abundance of *Dubosiella*, *Lactococcus*, *Enterorhabdus*, and *Ligilactobacillus* in the gut of HFD-fed mice [308]. Additionally, this drug increases the levels of *Lactobacillus*, a SCFA-producing bacteria, while reducing *Ruminococcus* and *Adlercreutzia*, changes that are associated with improved liver fibrosis in mice [343]. In T2D patients, empagliflozin also increases the abundance of SCFA-producing bacteria, including *Roseburia*, *Eubacterium*, and *Faecalibacterium*, and reduces several harmful bacteria such as *Escherichia-Shigella*, *Bilophila*, and *Hungatella* [344]. Dapaglifozin was observed to increase *Akkermansia muciniphila* levels in diabetic mice, being one of the potential mechanisms by which this drug reduces intestinal inflammation and promotes gut barrier integrity [345]. Additionally, dapagliflozin exerts a protective effect against renal inflammation and fibrosis in mice by decreasing harmful metabolites such as argininosuccinic acid and palmic acid, as well as the abundance of *Desulfovibrionaceae*, while increasing levels of *S*-allylcysteine and *Muribaculaceae* [346].

Collectively, these findings highlight the growing recognition that SGLT2 inhibitors exert clinically relevant effects beyond glucose control by reshaping the gut microbiome. Across multiple models, these drugs consistently increase beneficial SCFA-producing and barrier-supporting taxa while reducing pro-inflammatory or metabolically detrimental microbes and metabolites. Although research on the impact of ertugliflozin on the gut microbiota is limited, the recurring pattern of canagliflozin, empagliflozin, and dapagliflozin treatment is the restoration of a more anti-inflammatory and metabolically favorable microbial environment.

### 5.8. Anti-Inflammatories

Pro-inflammatory cytokines help the immune system respond to infections and tissue damage, but chronic low-grade inflammation, also referred to as inflammaging, is a key contributor to the aging process. Inflammaging is driven by a chronic activation of the immune system influenced by a range of cellular and molecular mechanisms, including decreased stem cell regeneration, impaired metabolism, loss of proteostasis, cellular stress, and epigenetic alterations [347]. In turn, inflammaging can further impair these processes, creating a self-reinforcing, bidirectional cycle and playing a key role in the pathogenesis of major age-related diseases, including T2D, cardiovascular diseases, Alzheimer’s disease, cancer, and other chronic conditions [348,349,350,351,352,353,354,355,356,357]. Age-related loss of skeletal muscle, commonly referred to as sarcopenia, has also become a major concern in the pursuit of healthy aging, as it is associated with frailty, reduced mobility, and increased risk of metabolic diseases [358,359]. Inflammatory cytokines inhibit muscle protein synthesis and promote muscle breakdown [360], suggesting that mitigating inflammation may help preserve muscle function and improve quality of life in older adults [361]. These findings raise the possibility that targeting chronic low-grade systemic inflammation could have therapeutic potential in the context of aging. While nonsteroidal anti-inflammatory drugs (NSAIDs) and corticosteroids pose serious long-term side effects, making them too risky for chronic use in older adults, more targeted therapies such as biologics, senolyics, and metformin offer a more feasible approach for addressing inflammaging. Since metformin and senolytics have already been discussed in this review, we will now focus on biologic anti-inflammatories, including TNF-a, IL-6, and IL-1 inhibitors. These compounds are commonly used to treat autoimmune diseases, severe asthma, and eczema, making them better candidates for long-term use [362,363,364,365].

#### 5.8.1. Mechanisms of Action of Anti-Inflammatories

**Figure 10:**
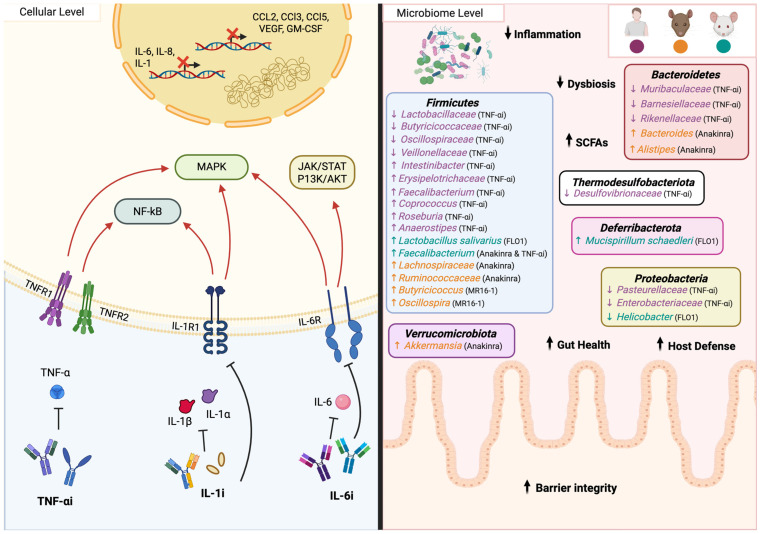
Overview of the pleiotropic effects of biological anti-inflammatories at the cellular and microbiome levels. Biological anti-inflammatory agents targeting TNF-α, IL-1, and IL-6 signaling inhibit receptor-mediated activation of key inflammatory pathways, including NF-κB, MAPK, JAK/STAT, and PI3K/AKT. This results in reduced transcription of pro-inflammatory cytokines and chemokines, attenuating inflammatory responses. Red lines indicate pathways inhibited by biologics. At the microbiome level, anti-inflammatory biologics reduce intestinal inflammation and dysbiosis, leading to increased SCFA-producing bacteria, enrichment of beneficial microorganisms, and reduction in pro-inflammatory pathobionts. Purple indicates gut microbiome changes in humans, orange in rats, and blue in mice. Created in BioRender. Medina-Cardena, M. (2026) https://BioRender.com/ywvqnp1.

Biologic anti-inflammatories are a class of targeted therapies that modulate the immune system by specifically inhibiting pro-inflammatory cytokines or their receptors, offering a powerful approach to control chronic inflammation (Figure 10). TNF-a (tumor necrosis factor alpha) is a central pro-inflammatory cytokine produced mainly by immune cells, and it is involved in the regulation of immune responses, inflammation, and tissue homeostasis by binding to the TNF1 and TNF2 receptors [366]. When TNF-a successfully binds to its receptors, it activates NF-κB and MAPKs with the help of TRAFs, increasing the production of inflammatory cytokines. Blocking TNF-a binding to TNF1 and TNF2 downregulates cytokine production and thus reducing chronic inflammation [367]. This can be achieved by monoclonal antibodies targeting TNF-a, such as adalimumab or infliximab, which bind directly to TNF-a and prevent it from activating the TNF1 and TNF2 receptors, or by TNF receptor fusion proteins such as etanercept, which acts as a decoy receptor by capturing TNF-a to block receptor activation before it can trigger inflammatory signaling [368]. The interleukin 1 (IL-1) family of cytokines is a potent pro-inflammatory cytokine family that regulates both innate and adaptive immunity by activating the gene expression of cytokines, chemokines, and adhesion molecules amplifying inflammation, being essential for immune responses against microbes and tissue damage [369]. Amongst its members, IL-1a and IL-1b are the most studied for autoinflammatory diseases [370]. Targeting IL-1 with biologics has proven effective in treating a broad spectrum of acute and chronic inflammatory conditions. This approach includes recombinant IL-1 receptor antagonists like anakinra, which binds to IL-1R1 to prevent IL-1a and IL-1b from activating downstream signaling; soluble decoy receptors such as rilonacept, which captures circulating IL-1 before it can reach cell surface receptors; and monoclonal antibodies like canakinumab, which specifically neutralizes IL-1b and block its pro-inflammatory effects [370]. Blocking IL-1 prevents the activation of NF-κB and key stress response members of the MAPK family such as JNK and p38, reducing the production of pro-inflammatory cytokines like IL-6, IL-8, and IL-1 itself. IL-6 binding to the IL-6R and gp130 forms a complex that recruits JAKs, which triggers downstream JAK/STAT3, PI3K/Akt, and MAPK pathways, leading to the transcription of genes involved in inflammation and angiogenesis, including IL 1β, IL-8, CCL2, CCL3, CCL5, GM-CSF, and VEGF [371]. IL-6 biologics include monoclonal antibodies against IL-6R such as tocilizumab and sarilumab, which block IL-6 binding and downstream signaling, as well as monoclonal antibodies that directly target IL-6, such as siltuximab, preventing activation of its receptor [372]. It is important to note that despite the promising results that anti-inflammatory biologics have shown, they are also associated with significant side effects that must be considered. For example, by targeting pro-inflammatory cytokines that play a role in immune defense, biologics can significantly increase the risk of infections by reducing the body’s ability to fight pathogens [373]. Currently, different biologics are being tested in clinical trials for the treatment of different diseases [362,374]. Although most of these therapies have been studied in the context of specific conditions rather than aging itself, they have been proposed as potential future treatments to address inflammaging [375].

#### 5.8.2. Impact of Anti-Inflammatories on the Gut Microbiota

Biologics primarily target host immune pathways rather than microbes directly; however, by reducing inflammation and restoring mucosal homeostasis, they can indirectly reshape gut microbiome composition and function (Figure 10) [376,377,378,379,380]. TNF-a inhibitors have been shown to restore the disrupted microbiome in spondyloarthritis (SpA) patients by decreasing the abundance of potentially pathogenic bacteria that are significantly upregulated in this disease, including *Muribaculaceae*, *Barnesiellaceae*, and *Rikenellaceae* from the *Bacteroidota* phylum; *Lactobacillaceae*, *Butyricicoccaceae*, *Oscillospiraceae*, and *Veillonellaceae* from *Firmicutes; Pasteurellaceae* and *Enterobacteriaceae* from *Proteobacteria*, and *Desulfovibrionaceae* from *Thermodesulfobacteriota* [376]. Conversely, taxa depleted in SpA, including *Intestinibacter* and *Erysipelotrichaceae*, increased following TNF inhibitor treatment [376]. Moreover, gut microbiota composition may be a key determinant of the onset and chronicity of inflammatory bowel disease (IBD) [381,382]. Treatment with TNF-a inhibitors has been associated with a shift toward a healthier gut microbiome, characterized by increased *Firmicutes*, particularly in butyrate-producing genera such as *Faecalibacterium*, *Coprococcus*, *Roseburia*, and *Anaerostipes*, which are generally linked to positive host health outcomes in this context [381]. Importantly, microbiome changes were observed to track with clinical response, suggesting that the gut microbiota may serve as a biomarker of therapeutic efficacy [383,384]. IL-1 inhibitors have been shown to beneficially modulate the gut microbiome in preclinical studies. Neutralization of IL-1α with FLO1, a monoclonal antibody used in animal studies, corrected mucosal dysbiosis and ameliorated ileitis in mice with IBD [378]. Specifically, FLO1 treatment decreased *Helicobacter* abundance and the *Proteobacteria* to *Firmicutes* ratio while increasing *Mucispirillum schaedleri* and *Lactobacillus salivarius*, bacteria associated with anti-inflammatory effects [378]. Experiments in germ-free mice demonstrated that the presence of a gut microbiome is essential for the beneficial effects of this IL-1α inhibitor [378]. In addition, pre-treatment of young mice with FLO1 protected them against experimentally induced colitis [378]. Overall, FLO1 treatment reduced inflammation and dysbiosis, reducing the severity of IBD in mice, and exerting anti-inflammatory effects that shift the gut microbiome toward a healthier, more beneficial composition. Similarly, anakinra, which is FDA-approved for the treatment of several inflammatory diseases, was shown to protect mice from both acute and chronic microbial injury, reduce mucosal barrier damage linked to dysbiosis, and limit the expansion of enteric pathogens [379]. Anakinra treatment promoted a more stable microbiota, including increased abundance of *Faecalibacterium*, bacteria associated with multiple beneficial effects on host health [379,385,386]. Moreover, it mildly increased the overall levels of *Firmicutes* and *Bacteroidetes* in mice and *Bacteroides*, *Alistipes*, *Lachnospiraceae*, *Ruminococcaceae* and *Akkermansia* in diabetic rats [379,387]. Finally, tocilizumab, an FDA-approved IL-6 inhibitor, has been associated with delayed gastrointestinal healing and an increased risk of bacterial translocation, septicemia, and disseminated candidiasis in patients treated with CAR-T cell therapy presenting immunosuppression and pre-existing GI-injury [388]. In contrast, preclinical studies using MR16-1, a preclinical rodent antibody blocking IL-6 signaling used to support tocilizumab’s approval, demonstrated protective effects on the gut microbiome [380,389]. MR16-1 restored *Firmicutes* levels and normalized the *Firmicutes*-to-*Bacteroidetes* ratio in stressed mice with depressive-like phenotypes, protected them against stress-induced dysbiosis, and increased butyrate-producing bacteria such as *Oscillospira* and *Butyricicoccus*, that suppresses inflammation, and provides energy to enterocytes [380]. Overall, biologics such as TNF-α, IL-1, and IL-6 inhibitors can indirectly reshape the gut microbiome by reducing inflammation and restoring mucosal homeostasis, promoting beneficial bacteria taxa linked to host health. Despite promising findings, data in humans remain limited, and the precise mechanisms of microbiome modulation are not fully understood. Key questions including long-term effects, patient-specific responses, and links to clinical outcomes remain to be addressed. Further clinical studies in humans using FDA-approved biologics are required to confirm these preclinical findings and to better understand how biologic anti-inflammatories influence the gut microbiome.

## 6. Discussion

**Figure 11:**
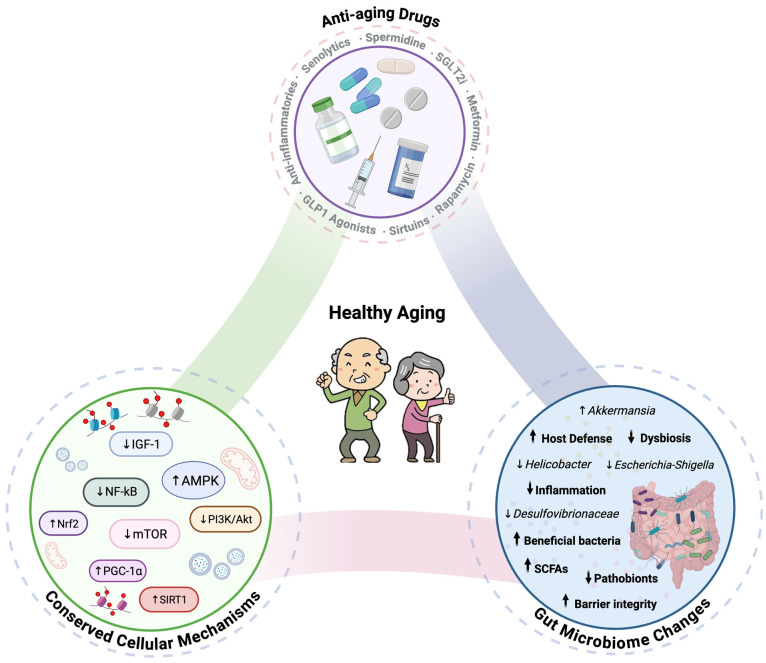
Crosstalk between anti-aging therapeutics, cellular mechanisms, and the gut microbiome in healthy aging. Created in BioRender. Medina-Cardena, M. (2026) https://BioRender.com/g1jmwky.

Despite targeting distinct molecular pathways, anti-aging interventions frequently converge on conserved regulatory systems involved in energy sensing, inflammation, and cellular stress responses, including AMPK, mTOR, SIRT1, autophagy, mitochondrial function, insulin/IGF-1 signaling, and NF-κB-mediated inflammation. Notably, modulation of the gut microbiome recurs across all pharmacological interventions, suggesting that host-microbe interactions may represent a common modulatory layer through which anti-aging therapies influence healthspan (Figure 11).

Building on this convergence, a closer examination of microbiome-associated changes reveals that, rather than inducing a uniform taxonomic signature, these therapies consistently promote microbial functions linked to metabolic and immune homeostasis. In particular, enrichment of SCFA-producing bacteria emerges as one of the most repeated microbial outcomes. This shift toward enhanced SCFA production is associated with anti-inflammatory effects, improved intestinal barrier integrity, enhanced mitochondria metabolism, and immune homeostasis [10,54], all processes closely linked to delayed functional decline during aging, although in certain contexts, elevated SCFA levels have been correlated with obesity [49,390]. In addition, metformin in specific metabolic contexts, senolytics, GLP-1R agonists, SGLT2 inhibitors, and certain anti-inflammatory therapies favor the increase in *Akkermansia*, which has been shown to improve epithelial integrity, enhance insulin sensitivity, and reduce chronic inflammation [34,39,391,392]. These findings further support the idea that anti-aging interventions promote microbiome configurations that reinforce gut barrier function and metabolic resilience. In parallel, many of the anti-aging interventions are associated with a reduction in potential pro-inflammatory bacterial taxa, including members of the *Desulfovibrionaceae* family, *Helicobacter*, and *Enterobacteriaceae* such as *Escherichia-Shigella*. Depletion of these bacteria aligns with decreased production of endotoxins and inflammatory metabolites, restoration of mucosal homeostasis, and suppression of chronic immune activation, all of which are central drivers of inflammaging [129,341,393,394,395].

Despite these shared patterns, a key unresolved question remains whether microbiome alterations actively mediate the beneficial effects of anti-aging drugs or predominantly reflect downstream consequences of improved host physiology. This distinction is complicated by the bidirectional nature of host-microbiome interactions and by strong context dependency. An important limitation of the current review is that microbiome signatures associated with anti-aging interventions show substantial variability across metabolic conditions and across experimental models. Such heterogeneity limits cross-study comparability and complicates the identification of common microbiome-mediated mechanisms. This challenge is further exacerbated by methodological differences among the included studies, particularly in the taxonomic resolution of microbiome analyses, with some studies reporting changes at the phylum level and others focusing on family-, genus-, or species- alterations. Moreover, many studies rely primarily on associative rather than mechanistic evidence, further constraining causal interpretation. Addressing these limitations will require mechanistic approaches capable of establishing causality, including microbiota-depleted and gnotobiotic models and microbiome-informed clinical trials, as well as greater standardization of key factors known to influence the gut microbiome, such as host age, sex, genetic background, diet, and treatment duration.

Summary of how anti-aging drugs promote healthy aging by modulating key cellular pathways to reduce inflammation, enhance autophagy, and stimulate mitochondrial biogenesis, while also improving microbiome balance by decreasing pro-inflammatory/pathobiont taxa, boosting SCFA production, and increasing the abundance of beneficial bacteria such as *Akkermansia*, which support gut barrier integrity and host defense.

## 7. Conclusions

The evidence reviewed indicates that, despite targeting different molecular pathways, many anti-aging interventions produce similar functional changes in the gut microbiome. Rather than inducing a single microbial profile, these interventions tend to promote a more resilient and adaptive gut ecosystem. While causality remains to be fully established, these shared microbiome-associated effects suggest that the gut microbiome may serve as an important interface through which different longevity-promoting strategies influence aging. Recognizing the potential active role of the microbiome could help refine therapeutic approaches and improve strategies aimed at promoting healthy aging.

## Data Availability

No new data were created or analyzed in this study. Data sharing is not applicable to this article.

## Abbreviations

The following abbreviations are used in this manuscript:

Aβ	Amyloid beta
AD	Alzheimer’s disease
AKT	Protein kinase B
AMPK	AMP-activated protein kinase
ATG	Autophagy-related genes
ATP	Adenosine triphosphate
BCL-2	B-cell lymphoma 2
BCL-xL	B-cell lymphoma–extra large
BCR-ABL	Breakpoint cluster region–Abelson
CAR-T	Chimeric antigen receptor T cell
CCL	Chemokine (C-C motif) ligand
CR	Caloric Restriction
cAMP	Cyclic adenosine monophosphate
CNS	Central nervous system
CXCL12	Stromal cell-derived factor 1
D	Dasatinib
DNA	Deoxyribonucleic acid
DPP-4	Dipeptidyl peptidase-4
eIF5A	Eukaryotic translation initiation factor 5A
EPACs	Exchange proteins directly activated by cAMP
EPHA2	EPH receptor A2
ER	Endoplasmic reticulum
FDA	Food and Drug Administration
FKBP12	FK506-binding protein 12 kDa
GABA	Gamma-aminobutyric acid
GI	Gastrointestinal
GIP	Glucose-dependent insulinotropic polypeptide
GLP-1	Glucagon-like peptide-1
GM-CSF	Granulocyte-macrophage colony-stimulating factor
gp130	Glycoprotein 130
GRAS	Generally recognized as safe
HFD	High-fat diet
IBD	Inflammatory bowel disease
IGF-1	Insulin-like growth factor 1
IL	Interleukin
IL-1	Interleukin-1
IL-1α	Interleukin-1 alpha
IL-1β	Interleukin-1 beta
IL-6	Interleukin-6
IL-6R	Interleukin-6 receptor
JAK	Janus kinase
JNK	c-Jun N-terminal kinase
MAMPs	Microbial-associated molecular patterns
MAPK	Mitogen-activated protein kinase
MMP3	Matrix metalloproteinase 3
mTOR	Mechanistic target of rapamycin
mTORC1	Mechanistic target of rapamycin complex 1
mTORC2	Mechanistic target of rapamycin complex 2
NF-κB	Nuclear factor kappa-light-chain-enhancer of activated B cells
NRF2	Nuclear factor erythroid 2–related factor 2
OCT	Organic cation transporters
p16	Tumor protein p16
p21	Tumor protein p21
p53	Tumor protein p53
PGC-1α	Peroxisome proliferator-activated receptor gamma coactivator 1-alpha
PI3K	Phosphoinositide 3-kinase
PKA	Protein kinase A
Q	Quercetin
RAP1	Ras-related protein 1
RNA	Ribonucleic acid
ROS	Reactive oxygen species
SASP	Senescence-associated secretory phenotype
SCFAs	Short-chain fatty acids
SFB	Segmented filamentous bacteria
SGLT2	Sodium-glucose cotransporter 2
STACs	Sirtuin activating compounds
SpA	Spondyloarthritis
SRC/SFKs	SRC family kinases
STAT3	Signal transducer and activator of transcription 3
TFEB	Transcription factor EB
TGF-β	Transforming growth factor beta
TLR4	Toll-like receptor 4
TMAO	Trimethylamine-N-oxide
TNF-α	Tumor necrosis factor alpha
TOR	Target of rapamycin
VEGF	Vascular endothelial growth factor
